# The Influence of Altered-Gravity on Bimanual Coordination: Retention and Transfer

**DOI:** 10.3389/fphys.2021.794705

**Published:** 2022-01-07

**Authors:** Ana Diaz-Artiles, Yiyu Wang, Madison M. Davis, Renee Abbott, Nathan Keller, Deanna M. Kennedy

**Affiliations:** ^1^Bioastronautics and Human Performance Lab, Department of Aerospace Engineering, Texas A&M University, College Station, TX, United States; ^2^Neuromuscular Coordination Lab, Department of Health and Kinesiology, Texas A&M University, College Station, TX, United States

**Keywords:** tilt paradigm, simulated microgravity, force control, Lissajous displays, motor learning

## Abstract

Many of the activities associated with spaceflight require individuals to coordinate actions between the limbs (e.g., controlling a rover, landing a spacecraft). However, research investigating the influence of gravity on bimanual coordination has been limited. The current experiment was designed to determine an individual’s ability to adapt to altered-gravity when performing a complex bimanual force coordination task, and to identify constraints that influence coordination dynamics in altered-gravity. A tilt table was used to simulate gravity on Earth [90° head-up tilt (HUT)] and microgravity [6° head-down tilt (HDT)]. Right limb dominant participants (*N* = 12) were required to produce 1:1 in-phase and 1:2 multi-frequency force patterns. Lissajous information was provided to guide performance. Participants performed 14, 20 s trials at 90° HUT (Earth). Following a 30-min rest period, participants performed, for each coordination pattern, two retention trials (Earth) followed by two transfer trials in simulated microgravity (6° HDT). Results indicated that participants were able to transfer their training performance during the Earth condition to the microgravity condition with no additional training. No differences between gravity conditions for measures associated with timing (interpeak interval ratio, phase angle slope ratio) were observed. However, despite the effective timing of the force pulses, there were differences in measures associated with force production (peak force, STD of peak force mean force). The results of this study suggest that Lissajous displays may help counteract manual control decrements observed during microgravity. Future work should continue to explore constraints that can facilitate or interfere with bimanual control performance in altered-gravity environments.

## Introduction

Numerous investigations over the past 50+ years have demonstrated significant detrimental effects associated with spaceflight, including sensorimotor function. Sensorimotor function is likely impaired by physiological adaptation to novel gravitational environments (Clark et al., 2015; De Sá Teixeira et al., 2017; Diaz Artiles et al., 2018; Galvan-Garza et al., 2018; Diaz-Artiles and Karmali, 2021; Goswami et al., 2021). Exposure to microgravity may, for example, cause sensorimotor discordance because our sensorimotor systems are calibrated to Earth’s gravity (Bock, 1998). Adapted changes in sensorimotor function during spaceflight, along with spatial disorientation and motion sickness (Lackner and DiZio, 2006; Diaz-Artiles et al., 2017), can result in poor manual control and coordination (Merfeld, 1996; Paloski et al., 2008). Such impairments may pose significant risks to operational tasks. Failure to complete mission tasks could have catastrophic consequences, resulting in loss of life, vehicle, or other property. Much of the research investigating manual control in altered-gravity has focused on unimanual performance (e.g., controlling joystick with dominant limb) (e.g., Clark et al., 2015; Clément et al., 2018; Rosenberg et al., 2018). However, many of the activities associated with spaceflight require individuals to use both limbs simultaneously (e.g., controlling a rover, landing a spacecraft).

Performing and learning bimanual tasks are different, and often more difficult, than unimanual tasks because individuals must control and coordinate actions for two limbs (Puttemans et al., 2005). Bimanual tasks are characterized by precise spatiotemporal relationships between the limbs and are described using variables that reflect the spatial and/or timing relationship between the limbs (e.g., relative phase, frequency relationship). For example, a relative phase value of 0° (in-phase) indicates that the two limbs are at the same point at the same time while a relative phase value of 90° indicates a quarter-cycle lag between the two limbs. Similarly, a 1:1 (in-phase) frequency relationship indicates that the limbs are synchronized in time and space while a 1:2 frequency relationship indicates that one limb is producing two actions for every one action produced by the contralateral limb. A large body of research has focused on how bimanual coordination patterns emerge, stabilize, and transition within Earth’s gravitational field (e.g., Kelso, 1984, 1994). Results have identified only two inherently stable bimanual coordination patterns: in-phase (0°) and antiphase (180°), with in-phase pattern being more stable than the antiphase pattern. Other phase (e.g., 90°) and frequency (e.g., 1:2) relationships have proved difficult or near impossible to perform and learn without significant training (e.g., Summers et al., 1993; Fontaine et al., 1997). It is not clear how gravity impacts bimanual coordination dynamics; however, gravity is known to alter the spatiotemporal structure of motor actions (Papaxanthis et al., 2005).

The attainment of bimanual tasks involves a process of motor learning (Newell, 1991), which is measured by analyzing task performance during acquisition, retention, and transfer (Magill and Anderson, 2013; Muratori et al., 2013). During acquisition (i.e., training), bimanual performance is often assessed using performance curves to identify how accuracy and/or stability evolves across training (e.g., Zanone and Kelso, 1992; Kelso and Zanone, 2002). The goal for a bimanual task is to increase performance accuracy and stability across trials. Learning, however, cannot be directly assessed and is typically inferred from retention and/or transfer tests (Magill and Anderson, 2013). Retention tests repeat the trained skill (e.g., 90°, 1:2) after a specified period (e.g., 15 min, 1 h, 1 day). Performance on the retention test is compared to performance at the end of training. If performance on the retention test is similar or better (e.g., more accurate, less variable) than performance at the end of training, then learning is inferred. Transfer tests present a task that is in some way novel (e.g., new coordination pattern, new environment) to determine if the skill practiced can be transferred to a new condition. Performance on the transfer test is then compared to performance at the end of training. If performance is similar or better, then learning is inferred. This classic motor learning paradigm was used, for example, in a bimanual control experiment, in which participants were trained to produce a complex 5:3 multi-frequency bimanual coordination task (Kovacs et al., 2010a). Following training, a 15-min retention interval was provided before the retention and transfer tests were administered. For the retention test, participants repeated the 5:3 multi-frequency bimanual coordination task and for the transfer test, participants were asked to produce a novel bimanual task with a similar level of difficulty (i.e., 4:3) (Kovacs et al., 2010a).

The difficulties associated with producing and learning bimanual tasks such as 90° relative phase and 1:2 multi-frequency relationships have been attributed to both inherent and incidental constraints (e.g., Swinnen, 2002). Inherent constraints are associated with the structure of the neuromuscular system (Carson and Kelso, 2004). For example, the control signals to each limb may be susceptible to the effects of neural crosstalk (Hoyer and Bastian, 2013; Kennedy et al., 2015). Neural crosstalk occurs during bimanual tasks when a mirror image of the motor command sent to one muscle group is also dispatched to the homologous muscles of the contralateral limb via crossed and uncrossed corticospinal pathways (Cattaert et al., 1999; Swinnen, 2002). As such, 1:1 in-phase bimanual tasks may be stabilized when complementary contralateral and ipsilateral signals are integrated, whereas other phase and frequency patterns may suffer from ongoing interference due to the conflicting information between the neural signals controlling the two limbs (Marteniuk et al., 1984; Maki et al., 2008). Because 1:1 in-phase is an inherently stable bimanual coordination pattern, researchers often compare performance of the to-be-learned coordination pattern (e.g., 1:2) to the 1:1 in-phase task (e.g., Kovacs and Shea, 2011; Herth et al., 2021). This comparison may provide additional clues regarding constraints associated with performing and learning of complex bimanual tasks. Research has also indicated that the effects of neural crosstalk is partially dependent on the force requirements of the task, with higher forces resulting in stronger crosstalk effects, and lower forces in weaker crosstalk effects (Heuer et al., 2001; Kennedy et al., 2017). As such, it is possible that gravitational force acting on the body may influence an individual’s ability to effectively produce and learn bimanual tasks.

Alternatively, incidental constraints are associated with specific perceptual, cognitive, and/or attentional features of the task or task environment (Shea et al., 2016). A number of investigations have provided compelling support for the notion that inherent constraints govern bimanual coordination dynamics (Mechsner et al., 2001; Li et al., 2004; Mechsner and Knoblich, 2004). For example, research has demonstrated that complex coordination patterns, once thought difficult or near impossible to perform without significant practice, could be performed within a few minutes of practice with relatively simple feedback manipulations (e.g., Kovacs et al., 2020; Wang et al., 2021). In these experiments, the feedback manipulations provided real-time information that integrated multiple sources of information into a simplified output display using mathematically generated Lissajous plots and movement templates (Shea et al., 2016). Lissajous plots integrate the position of two limbs into a single point (cursor) in one plane, with one limb moving the cursor in the horizontal direction while the other limb moves the cursor in the vertical direction. Lissajous displays have been used to successfully produce bimanual coordination patterns with relative phases from 0 to 180° (Kovacs and Shea, 2011; Kovacs et al., 2020), multi-frequency ratios (Kovacs et al., 2010a,b; Kennedy et al., 2016a), asymmetric amplitudes (Kovacs and Shea, 2011), asymmetric forces (Kennedy et al., 2017), different task goals for each limb (Wang et al., 2013), continuous transitions through the attractor landscape (Kennedy et al., 2016b), and intermanual patterns where two different people controlled the cursor (Kovacs et al., 2020; Wang et al., 2021). The ability to perform complex multi-frequency bimanual tasks within a few minutes of training when provided Lissajous displays is quite remarkable when compared to previous research that provided up to 8 days of training during acquisition to produce the goal coordination patterns (e.g., Summers et al., 1993).

Recently, Kovacs et al. (2020) directly compared coordination performance between participants who were provided either (1) Lissajous plots or (2) traditional pacing metronomes to produce bimanual coordination patterns between 0 to 180° relative phase. The results indicated that participants were quite effective (low error and variability) at producing the goal coordination patterns within a few minutes of practice when they were provided Lissajous plots, whereas the same complex patterns (30–150°) were not performed well (high error and variability) when participants were provided metronomes. These results point to incidental constraints related to the difficulty in producing complex bimanual coordination patterns rather than interference associated with neural crosstalk.

The integrated feedback information provided by the Lissajous plots likely reduced the attentional, cognitive, and/or perceptual constraints associated with task performance (Shea et al., 2016). However, given the increased attentional, cognitive, and perceptual demands associated with spaceflight and altered-gravity environments (e.g., Saradjian et al., 2014; Friedl-Werner et al., 2021), it is not clear whether integrated feedback information can be used to perform and learn complex bimanual tasks in microgravity, similar to that observed in normal gravity (1 g). In addition, astronauts train for operational tasks in a 1 g environment; therefore, understanding constraints that influence performance and learning in 1 g environment, and how these constraints transfer to novel gravity environments may have important implications for future training protocols and countermeasures. Therefore, the purpose of the current investigation was to determine if participants can effectively produce and learn a complex bimanual coordination task when provided Lissajous plots, and to examine the inherent and incidental constraints acting on the system during the learning and transfer process to simulated microgravity.

## Materials and Methods

### Participants

Twelve young adults (Mean age ± *SD* = 21.9 ± 3.03; 6 females and 6 males) volunteered to participate in the experiment. Due to performance asymmetries associated with limb dominance during bimanual tasks (e.g., Cattaert et al., 1999; Aramaki et al., 2006; Kennedy et al., 2017), only right limb dominant participants were included. Limb dominance was confirmed with a standardized survey (Oldfield, 1971). Participants read and signed a consent form approved by the Texas A&M University Institutional Review Board for the ethical treatment of experimental participants, in accordance with the 1964 Helsinki Declaration and its latest 2013 amendment. Subjects were not informed of the specific purpose of the study and none of the subjects had prior experience performing the experimental tasks.

### Apparatus

**FIGURE 1:**
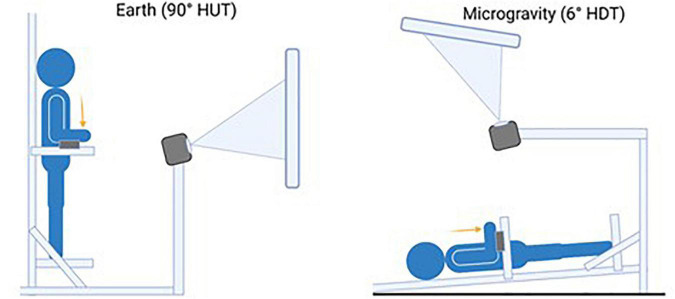
Illustration depicting the apparatus and testing positions to simulate the two gravitational loads in the head-to-toe direction (Gz axis): (Left) Earth [upright position or 90° Head Up Tilt (HUT)]; (Right) Microgravity [6° Head Down Tilt (HDT)]. In each condition, a projector screen is positioned in front of the subjects at their eye level.

A custom aluminum tilting platform designed to simulate a variety of gravitational loads in the head-to-toe direction (i.e., Gz axis) was used for this experiment, and it is shown in Figure 1. This tilt paradigm is commonly used to simulate altered-gravity conditions (Petersen et al., 2021; Whittle et al., 2021). Two gravity levels were simulated by tilting the platform to the designated angle: Earth [90° Head Up Tilt (HUT)] and microgravity [6° Head Down Tilt (HDT)]. Specifically, the 6° HDT position is a widely accepted analog for microgravity conditions (Diaz-Artiles et al., 2019). Force transducers were fixed in an adjustable fashion such that subjects’ arms would be at their side and elbows bent 90° with forearms resting against the force transducers. A projector was mounted in front of the custom tilt table with a projector screen fixed at the subject’s eye level.

### Bimanual Task: Force Coordination

**FIGURE 2:**
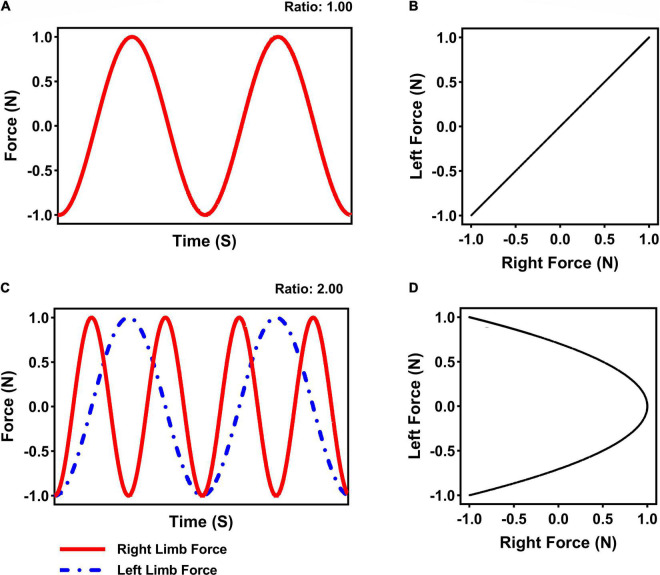
Illustration depicting the goal 1:1 in-phase **(A)** and 1:2 multi-frequency **(C)** limb time series produced using the Lissajous displays for the 1:1 **(B)** and 1:2 **(D)** bimanual coordination task.

Participants were instructed to rhythmically produce specific patterns of isometric forces with both forearms against the force transducers. Specifically, individuals applied force on the left-side transducer with their left forearm and on the right-side transducer with the right forearm in coordinated patterns: 1:1 (i.e., in-phase: limbs produced a pattern of force simultaneously) or 1:2 (i.e., multi-frequency: right limb produced 2 patterns of force for every 1 pattern for the left limb) frequency ratios using a visual guide in the form of a Lissajous displays (see Shea et al., 2016). These Lissajous displays provided integrated visual feedback regarding limbs force production as one point (cursor) in a single plane. Thus, applying force with the left limb moved the cursor from the bottom of the display to the top of the display, whereas applying force with the right limb moved the cursor from the left side of the display to right side of the display. The Lissajous displays were presented to participants along with a goal template to produce the force coordination patterns of 1:1 or 1:2 (see Figures 2B,D; only the goal templates were available to subjects. The cursor was also projected on the screen).

### Experimental Design

**FIGURE 3:**
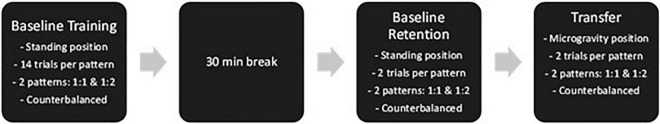
Summary of the experimental design.

Baseline data collection (also considered baseline training) was conducted in upright position (Earth condition: 90° HUT). Participants performed 14 trials of each force coordination task (1:1 and 1:2 pattern, in a counterbalance order, making a total of 28 training trials). Each trial was 20 s long with 10 s in between trials. After a 30-min break, participants performed a baseline retention test, consisting of 2 trials of each coordination pattern (1:1 and 1:2 frequency ratio, in a counterbalance order, making a total of 4 baseline retention trials) in upright position (Earth condition: 90° HUT). Then, transfer tests were conducted where participants performed 2 additional trials, of each coordination pattern, in the microgravity condition. The order of coordination patterns (1:1 and 1:2) was counterbalanced. The experimental design is summarized in Figure 3.

### Dependent Measures

Data collection and reduction was conducted using MATLAB (v2020a, The MathWorks, Inc., Natick, MA). Limb forces were filtered using a second order dual pass Butterworth filter with a cutoff frequency set at 10 Hz. A 3-point difference algorithm was used to compute force velocity and force acceleration. Force production measures were further detrended and normalized between −1 and 1 (see Figure 2). Based on these measures, the following dependent measures were calculated and further analyzed:

– *Unimanual Measures*: Interpeak interval (IPI), standard deviation of the interpeak intervals (STDIPI), phase angle velocity, peak force, standard deviation of peak force (STD peak force), and mean force.
– *Bimanual Measures*: Interpeak interval ratio (IPI ratio), phase angle slope ratio, and interpeak interval ratio error (IPI ratio error).

Further details on how these measures were extracted and analyzed are provided below.

#### Unimanual Measures

Interpeak intervals (IPI) represent the time between two consecutive force peaks The values were computed for each limb on a cycle-by-cycle basis with each cycle representing every other zero crossing of the force signal. In addition, the standard deviation (STD) of the interpeak intervals (STDIPI) provides information regarding the IPI variability for each limb and was determined using the standard deviation of the interpeak intervals within a trial.

Phase angle velocity provides information regarding the rate at which each force pulse was produced. This measure was calculated for each limb by first normalizing the force time series. Thus, the force time series were mean-centered around zero and then amplitude rescaling was performed on a half-cycle basis by dividing the positive and negative amplitudes with their corresponding peak positive and peak negative amplitude scores. Next, the phase angle (ϕ) for each limb (*i* = r, l) was computed for the normalized force time series as follows (Kelso et al., 1986):


$$\phi =\tan^{-1}\,\left[\left({\text{dX}}_{i}/\text{d}\,t\right)/{\text{X}}_{i}\right]$$

where X*_*i*_* represents the normalized force of the right and left limbs and dX*_*i*_*/d*t* the instantaneous normalized force velocities for each limb. Next, the individual phase angles ϕ were unwrapped by finding absolute jumps greater than 2π and adding appropriate multiples of 2π to each data point following the jump. A 3-point difference algorithm was then used to compute phase angle velocity. During the 1:1 task, the phase angle velocity should be the same for each limb whereas in the 1:2 task, the phase angle velocity should be roughly twice as fast for the right limb as the left limb.

Peak force, STD of peak force, and mean force were calculated to determine the control of force for each limb. Peak force was calculated by averaging peak forces across the trial. The STD peak force was defined as the standard deviation of all identified peak forces across the trial. Mean force was calculated by averaging the absolute force produced during each trial. All three measures were calculated for both the left and right limb. Note the goal coordination pattern required a peak force of 30 N and a mean force of 15 N.

#### Bimanual Measures

IPI ratio provides a temporal measure of goal attainment that is independent of limb coordination tendencies and actual limb force trajectories. IPI ratio uses interpeak intervals for the right and left limb to determine point estimates of mean cycle duration and compute a frequency ratio of left limb cycle duration to right limb cycle duration. An IPI ratio of 1.0 indicates that the interval for the right and left limb are equal while an IPI ratio of 2.0 indicates that the interval for the left limb is twice that of the right limb.

IPI ratio error was used to quantify deviations from the goal IPI ratio. IPI ratio error was calculated by subtracting performed IPI ratio from the goal IPI ratio (1.0 for the 1:1 task and 2.0 for the 1:2 task). IPI ratio error was calculated for each trial (training, retention, and transfer).

Phase angle velocity ratio provides a continuous measure of bimanual goal attainment. It uses continuous phase angles for the two limbs to examine the continuous spatial-temporal coordination of limb forces. To determine phase angle velocity ratio, a regression analysis of the continuous relative phase velocities for each limb were conducted to calculate the slope of the unwrapped right limb phase angle velocity to left limb phase angle velocity across the trial for each participant. Similar to IPI ratio, the goal phase angle velocity ratio for the 1:1 task would be 1.0 while the goal phase angle velocity ratio for the 1:2 task would be 2.0.

### Data Analysis and Statistics

#### Learning, Retention, and Transfer

The IPI ratio error was calculated across the training trials for the two bimanual coordination tasks (1:1 and 1:2 tasks). This includes, for each coordination task, the first 14 training trials in Earth condition, retention of Earth condition, and transfer to microgravity conditions (coordination tasks presented in counterbalanced order across subjects).

Data did not satisfy the normality assumption required to conduct parametric testing. Thus, a non-parametric Friedman test for dependent samples was implemented to investigate the “learning, retention, and transfer effects,” which we will refer to as “time” effects, in both 1:1 and 1:2 coordination tasks (1:1, 1:2). Thus, four specific “time” points were included in the statistical analysis: the 2nd training trial or baseline (Earth condition), the 14th training trial (Earth condition), the best of the two retention trials (Earth condition), and the best of the two transfer trials (microgravity condition). To further analyze the “time” effects, *post hoc* analysis with a Wilcoxon signed-rank test with Bonferroni correction was conducted between the different time-points considered.

#### Unimanual and Bimanual Measures

The unimanual measures interpeak interval (IPI), phase angle velocity, and STD peak force, were analyzed in a repeated measures three-way ANOVA to investigate the influence of coordination task (1:1, 1:2), limb (left, right), and gravity level (Earth, microgravity). Simple main effects were also implemented to further investigate significant interaction effects. Data from these variables did not present any outliers and residuals were normally distributed, thus satisfying the required assumptions for this type of parametric testing. However, the rest of the unimanual measures [standard deviation of the interpeak intervals (STDIPI), peak force, and mean force] did not satisfy the normality assumption. Thus, a non-parametric Wilcoxon signed-rank test was implemented instead. Bimanual measures (interpeak interval ratio and phase angle slope ratio) were also analyzed using a non-parametric Wilcoxon signed-rank test to investigate the influence of coordination task (1:1, 1:2), and gravity level (Earth, microgravity).

All statistical tests were performed with IBM SPSS Statistics 25 software (IBM Corporation) and the significance level was set at α = 0.05. All data is presented as mean ± standard error (SE).

## Results

### Learning, Retention, and Transfer

**FIGURE 4:**
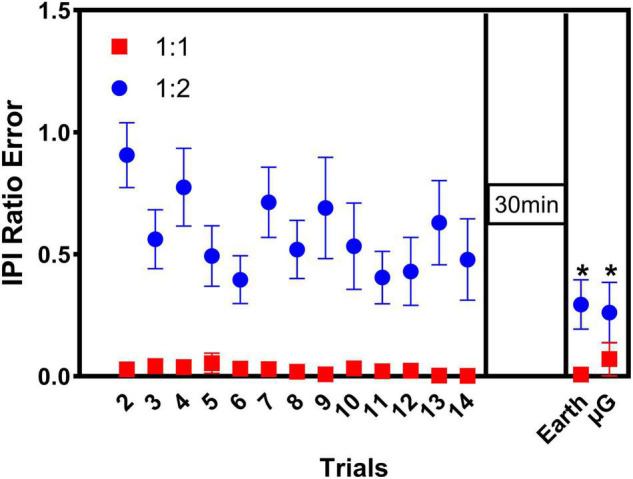
Interpeak interval (IPI) ratio error over time for both coordination tasks (1:1 and 1:2). Participants (*n* = 12) performed 14 trials of each coordination task (baseline training) in the upright position (Earth condition), followed by a 30 min break. Then, participants performed a retention test consisting in 2 additional trials, per coordination task, in the upright position. Finally, participants performed a transfer test, consisting in 2 additional trials, per coordination task, in microgravity conditions. Tasks were presented in counterbalanced order among participants. Only the best retention and transfer trial were included in the analysis. Statistically significant differences over time were found in the 1:2 task only: compared to baseline (2nd training trial), IPI ratio error was significantly smaller in retention trial (Earth) and transfer trial (microgravity). Significance: *p < 0.05. Data are presented as average ± SE.

Figure 4 shows the interpeak interval (IPI) ratio error over time for both coordination tasks (1:1 and 1:2). Thus, for each coordination task, the figure shows the IPI ratio error over the initial 14 training trials in upright position (Earth Condition). Then, after 30 min break, participants performed two retention trials in upright position (Earth condition), followed by two transfer trials in 6° HDT position (microgravity condition). For each subject, the best of the two retention trials and the best of the two transfer trials were selected for analysis and thus, only those data are included in Figure 4.

The statistical analysis indicated main effects of task [χ^2^(1) = 12.0, *p* < 0.022], where task 1:2 presented larger IPI Ratio errors than task 1:1. For the 1:1 task, the Friedman test indicated no significant change in IPI ratio errors over time [χ^2^(3) = 4.881, *p* = 0.181], which were already very small in the first place. However, for the 1:2 task, the Friedman test showed a significant improvement in performance (i.e., decrease in IPI ratio error) over time [χ^2^(3) = 12.9, *p* = 0.005]. *Post hoc* analysis with Wilcoxon signed-rank tests (with Bonferroni correction) indicated that, compared to baseline (i.e., 2nd training trial), IPI ratio error was significantly smaller at the retention trial in Earth condition (*Z* = −2.981, *p* = 0.015), and the transfer trial in microgravity condition (*Z* = −2.746, *p* = 0.030). The IPI ratio error from the retention trial (in Earth condition) and the transfer trial (in microgravity condition) were not statistically different from the 14th training trial (i.e., the last training trial). In addition, further analysis also indicated significant differences in IPI ratio errors between the 1:1 task and the 1:2 task at all-time points investigated (2nd training trial: *p* = 0.008; 14th training trial: *p* = 0.008; retention test in Earth condition: *p* = 0.008), except for the final transfer test in microgravity conditions (*p* = 0.092), suggesting that, at this point, participants performing the more complex 1:2 task were able to reach a level of performance comparable to the performance achieved during the easier 1:1 coordination pattern.

### Bimanual Results

**FIGURE 5:**
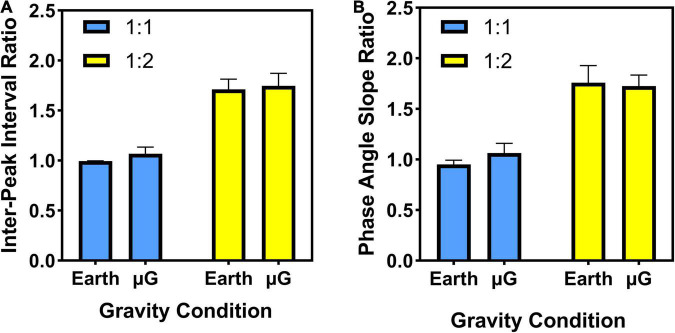
Bimanual measures: **(A)** Interpeak interval (IPI) ratio and **(B)** phase angle slope ratio. Coordination patterns: 1:1 in-phase and 1:2 multi-phase. For both bimanual measures, the goal value of 1.0 would indicate that both limbs were producing force pulses at the same rate (i.e., goal of the 1:1 coordination pattern), whereas a goal value of 2.0 would indicate that the right limb is producing two patterns of force for every one pattern of force produced by the left limb (i.e., goal of the 1:2 coordination pattern). Data are presented as average ± SE.

Figure 5 shows the bimanual dependent variables included in the analysis: interpeak interval ratio and phase angle slope ratio. Data are presented by coordination pattern (1:1 or 1:2) at the different simulated gravitational environments (Earth, microgravity).

#### Interpeak Interval Ratio

As expected, the statistical analysis revealed a main effect of task (*Z* = −2.981, *p* = 0.003), where the IPI ratio for the 1:1 coordination pattern (1.03 ± 0.03) is smaller than the IPI ratio of the 1:2 coordination pattern (1.73 ± 0.11). We note that the goal IPI ratio for the 1:1 and 1:2 tasks are 1.0 and 2.0, respectively, with no variability. Main effects of gravity (*Z* = −1.569, *p* = 0.117) were not significant. Taken together, these results indicate that subjects were very effective in producing the desired coordination pattern (1:1 and 1:2) at both gravity levels.

#### Phase Angle Slope Ratio

The statistical analysis indicated a main effect of task (*Z* = −3.059, *p* = 0.002), where the ratio for the 1:1 coordination pattern (1.01 ± 0.06) is smaller than the ratio of the 1:2 coordination pattern (1.74 ± 0.13). Similar to the results from IPI ratio, we note that the goal phase angle slope ratio for the 1:1 and 1:2 tasks are 1.0 and 2.0, respectively, with no variability. Main effects of gravity (*Z* = −0.235, *p* = 0.814) were not significant. These results also indicate that, based on regression analysis of the continuous phase angle data for both left and right limbs, subjects were very effective producing the goal coordination patterns (1:1 and 1:2) at both gravity levels.

### Unimanual Results

**FIGURE 6:**
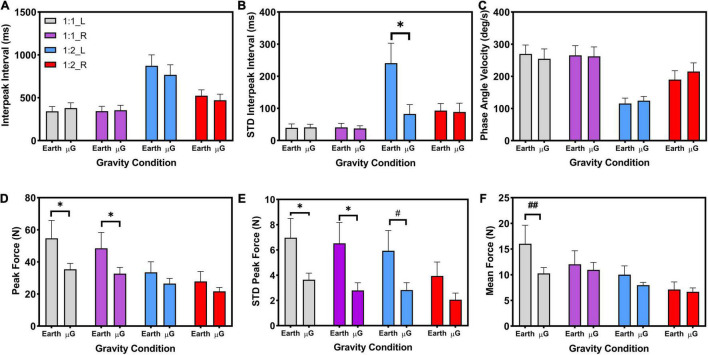
Unimanual measures: **(A)** Interpeak interval (ms), **(B)** Standard deviation of the interpeak interval (STDIPI) (ms) **(C)** Phase angle velocity (deg/s), **(D)** Peak force (N), **(E)** Standard deviation of the peak force (N), **(F)** Mean force (N). Coordination patterns: 1:1 and 1:2. Limbs: Left (L), and Right (R). Significance: *p < 0.05. Marginal significance: ^#^*p* = 0.058, ^##^*p* = 0.050. Data are presented as average ± SE.

Figure 6 shows the unimanual dependent variables included in the analysis: interpeak interval, standard deviation of interpeak interval, phase angle velocity, peak force, STD peak force, and mean force. Data are presented by coordination pattern (1:1 or 1:2), as well as by limb (L: left, R: right) at the different simulated gravitational environments (Earth, microgravity).

#### Interpeak Interval

The analysis indicated a main effect of task [*F*(1, 11) = 35.87, *p* < 0.001, η^2^*_*p*_* = 0.77], limb [*F*(1, 11) = 18.94, *p* = 0.001, η^2^*_*p*_* = 0.63], and task*limb interaction [*F*(1, 11) = 18.61, *p* = 0.001, η^2^*_*p*_* = 0.63]. All other interaction effects were not statistically significant. For the 1:1 task, simple main effects analysis indicated no significant change in IPI between the left and right limb. However, for the 1:2 task, simple main effects indicated a statistically significant change in IPI between the left and right limb (*p* = 0.001), where the left limb presents slower IPI than the right limb. In addition, for both left and right limbs, simple main effects also indicated that the 1:2 task was executed with higher IPIs (i.e., slower movement), than the 1:1 task (left limb: *p* < 0.001; right limb: *p* < 0.004).

#### Standard Deviation Interpeak Interval

The analysis indicated a main effect of task (*Z* = −2.903, *p* = 0.004), and a marginal effect of limb (*Z* = −1.961, *p* = 0.05). Simple main effects analysis indicated similar STD interpeak intervals for the 1:1 task, for the left and right limb, at any of the two gravity levels (i.e., Earth and microgravity). However, the left limb STD interpeak interval in Earth condition during the 1:2 task was statistically significantly higher than the same left limb STD interpeak interval during the same 1:2 task in the microgravity condition (*Z* = −2.045, *p* = 0.041). In addition, the left limb STD interpeak interval in Earth condition during the 1:2 task was statistically significantly higher than the right limb STD interpeak interval in those same Earth conditions during the 1:2 task (*Z* = −2.589, *p* = 0.010). Simple main effects also showed significantly larger STD interpeak intervals in Earth condition during the 1:2 task with respect to the 1:1 task for both the left limb (*Z* = −2.667, *p* = 0.008) and the right limb (*Z* = −2.353, *p* = 0.019).

#### Phase Angle Velocity

The analysis indicated a main effect of task [*F*(1, 11) = 41.65, *p* < 0.001, η^2^*_*p*_* = 0.79], limb [*F*(1, 11) = 26.37, *p* < 0.001, η^2^*_*p*_* = 0.71], task* limb interaction [*F*(1, 11) = 29.12, *p* < 0.001, η^2^*_*p*_* = 0.73], and limb*gravity interaction [*F*(1, 11) = 5.26, *p* = 0.043, η^2^*_*p*_* = 0.32]. All other interaction effects were not statistically significant. Simple main effects analysis indicated significant differences between the left and right limb in the Earth condition (*p* = 0.002) and in the microgravity condition (*p* < 0.001). Simple main effects analysis also indicated faster phase angle velocities during the 1:1 task with respect to the 1:2 task, for both left (*p* < 0.001) and right limb (*p* = 0.005).

#### Peak Force

The analysis indicated a main effect of task (*Z* = −3.059, *p* = 0.002), limb (*Z* = −2.510, *p* = 0.002), and gravity (*Z* = −2.197 = 0.028). Further simple main analysis during the 1:1 task indicated a significantly higher peak force in the Earth condition compared to the microgravity condition, for both left (*Z* = −2.197, *p* = 0.028) and right limb (*Z* = −2.432, *p* = 0.015).

#### Standard Deviation Peak Force

The analysis indicated a main effect of gravity [*F*(1, 11) = 11.19, *p* = 0.007, η^2^*_*p*_* = 0.50], where the STD peak force was significantly smaller on microgravity condition with respect to Earth condition. Results also indicated a main effect of limb [*F*(1, 11) = 8.634, *p* = 0.013, η^2^*_*p*_* = 0.44], where the left limb presented a significant higher STD peak force compared to the right limb. All interaction effects were not statistically significant. For the microgravity condition, simple main effects analysis indicated a significant higher STD peak force for the 1:1 task with respect to the 1:2 task (*p* = 0.024). Additionally, for the 1:1 task, simple main effects analysis also indicated a significantly higher STD peak force in the Earth condition with respect to the microgravity condition for both left limb (*p* = 0.022) and right limb (*p* = 0.009). For the 1:2 task, left limb STS peak force differences between Earth and microgravity conditions are marginally significant (*p* = 0.058, noted as # in Figure 6).

#### Mean Force

The analysis showed a main effect of task (*Z* = −2.824, *p* = 0.005), and limb (*Z* = −2.353, *p* = 0.019), indicating higher mean force during the 1:1 task with respect to the 1:2 task, and higher mean force in the left limb with respect to the right limb. Results did not indicate a main effect of gravity (*Z* = −1,020, *p* = 0.308). However, simple main effects analysis did indicate a marginal significant difference in mean force in the left limb during the 1:1 task between Earth and microgravity conditions (*Z* = −1.961, *p* = 0.050) (noted as a ## in Figure 6).

## Discussion

### Bimanual Coordination

Based upon two independent temporal measures of bimanual goal attainment (IPI ratio and phase angle velocity ratio, shown in Figure 5), results indicated that participants could effectively perform the 1:1 in-phase and 1:2 multi-frequency tasks in both the Earth and microgravity conditions. The performance curve (see Figure 4) clearly displays that performance accuracy (i.e., IPI ratio error) improved across training for the 1:2 task. This result replicates a number of ground studies demonstrating that participants can quickly and effectively perform a variety of complex coordination tasks when provided Lissajous display information (see Shea et al., 2016 for a review). The Lissajous displays provided goal templates for the 1:1 and 1:2 bimanual force tasks along with real-time feedback information regarding the pattern of force produced by the two limbs as a single point. Participants were able to use this information, regardless of the gravity condition, to produce the goal pattern. The ability to produce bimanual multi-frequency patterns within a few minutes of practice is quite impressive when compared to previous experiments in which multiple days of training were needed to successfully produce novel coordination patterns when metronomes were used to pace performance (e.g., Zanone and Kelso, 1992; Summers et al., 1993; Kelso and Zanone, 2002). Extending this line of research to altered-gravity environments provides further evidence for the robust utility of Lissajous displays in facilitating complex bimanual coordination tasks (Shea et al., 2016). Considering the increased attentional, cognitive, and perceptual demands associated with altered-gravity environments (Friedl-Werner et al., 2021), the ability to quickly and effectively produce a complex pattern of force in altered-gravity is particularly impressive.

For the 1:1 task, however, performance was remarkably accurate from the beginning of training. This result is consistent with a number of investigations indicating that the 1:1 in-phase pattern is inherently stable and easy to produce, while other phase and frequency patterns are less stable and difficult to perform without extended training (e.g., Byblow et al., 1994, 1998; Kelso, 1994; Puttemans et al., 2005) or feedback manipulations to reduce the demands of the task (Shea et al., 2016). Similar results were also observed in a recent experiment examining the effects of gravity on muscle synergies in arm cycling (Botzheim et al., 2021). Participants performed an arm cycling task in a supine or upright body position while cranking on small (radius = 10 cm) or large (radius = 15 cm) ergometers, using two different coordination modes (synchronous and asynchronous). It is important to note that the synchronous mode required in-phase coordination while the asynchronous mode required antiphase coordination. The results indicated that muscle coordination was affected by gravity and cranking mode, but not movement size. In terms of cranking mode, results indicated that muscle coordination was significantly higher when cycling in a synchronous mode. Interestingly, the authors suggested that this result was counterintuitive because asynchronous cycling is the common and usual mode of cycling (Botzheim et al., 2021). However, from a dynamical systems perspective, the cranking mode (synchronous/asynchronous, in-phase/antiphase) results observed by Botzheim et al. (2021) would be predicted by the Haken, Kelso, Bunz (HKB) model.

The HKB model provides a formal description of the stability properties associated with bimanual coordination in 1 g based on non-linear dynamics (Haken et al., 1985). In-phase and antiphase coordination patterns are represented as stable fixed-point attractors in the coordination landscape, with in-phase more stable than antiphase. Other coordination patterns (e.g., 90°, 1:2) represent repellers in the attractor landscape. A repeller drives a variable away from it and toward the attractor (e.g., in-phase coordination pattern). As such, spontaneous phase transitions to in-phase coordination patterns may disrupt all other bimanual coordination patterns (e.g., antiphase, 1:2) when the control parameter (e.g., frequency) is increased (e.g., Peper et al., 1995; Treffner and Turvey, 1996). However, it is not clear how gravity affects coordination dynamics. In terms of the temporal constraints associated with bimanual coordination, the results of the current investigation, as well as those by Botzheim et al. (2021), suggest that bimanual performance in altered-gravity environments is constrained by the same dynamical entrainment processes as on Earth.

Despite the effective timing of the isometric force pulses, however, differences in measures associated with force production (peak force, peak force variability, and mean force: Figures 6D,E,F). were observed between the Earth and microgravity conditions. More specifically, the results indicated higher peak force for both limbs during the 1:1 task for the Earth condition compared to the microgravity condition (Figure 6D). This finding contrasts with previous research that has reported exaggerated peak forces during manual control tasks performed in altered-gravity environments (Bock and Cheung, 1998; Sand et al., 2003; Mierau et al., 2008; Dalecki et al., 2012). However, it may be important to note that the majority of the tasks in these studies involved unimanual rather than bimanual control. Dalecki et al. (2012) did require participants to use two limbs to complete their task, but one limb was performing a four-choice reaction task by pushing a button while the contralateral limb was performing the manual control task using a joystick. Nevertheless, their results indicated higher peak isometric forces with simulated weightlessness by water immersion compared to the land (Earth) condition. The observed exaggerated peak isometric forces in these studies were attributed to degraded proprioceptive feedback mechanisms in non-normative gravity environments (e.g., hyper-gravity, microgravity, simulated weightlessness) (Bock and Cheung, 1998; Sand et al., 2003; Mierau et al., 2008; Dalecki et al., 2012). The results of the present study, however, found reduced peak forces in the microgravity condition compared to the Earth condition. The same pattern of results was also observed for peak force variability (Figure 6E).

It is possible that the differential peak force results between the current experiment and previous investigations using altered-gravity environments could be a function of task type (unimanual vs. bimanual; symmetric vs. asymmetric), and/or the role of proprioception in the neural control of bimanual actions. For example, previous research using deafferented patients to determine the role of proprioception in bimanual control indicated proprioception is not critical for achieving temporal coupling between the hands, nor does it contribute significantly to the disruption of asymmetric (e.g., antiphase) coordination (Spencer et al., 2005). Note, however, other investigations have reported that the timing of bimanual tasks is controlled by proprioceptive information (e.g., Verschueren et al., 1999). Similarly, research using stroke patients indicated that greater proprioceptive deficits were associated with deficits in symmetric (in-phase) coordination but not with asymmetric coordination (Kantak et al., 2016). Given that microgravity results in reduced and/or distorted proprioception (Kanas and Manzey, 2008), the results of the current investigation had a similar pattern to the investigations using patient populations with impaired proprioception. That is, differences in peak force were observed between the microgravity and Earth conditions for the symmetric 1:1 in-phase task but not for the more complex asymmetric 1:2 multi-frequency pattern. This may indicate deficits associated with feedforward motor command during 1:1 in-phase coordination in microgravity. That is, 1:1 in-phase is thought to be stabilized, at least in part, to interactions between feed-forward motor commands as the results of shared neural pathways (e.g., neural crosstalk) (Helmuth and Ivry, 1996; Ivry and Richardson, 2002; Ridderikhoff et al., 2005).

Neural crosstalk occurs when both hemispheres send motor commands to the contralateral limb via the crossed corticospinal pathways while simultaneously sending a mirror image of the motor command to the ipsilateral limb via the uncrossed corticospinal pathways (Cattaert et al., 1999; Cardoso de Oliveira, 2002). This ipsilateral influence may alter the activation of the involved muscle (Cattaert et al., 1999; Swinnen, 2002) resulting in increased or decreased contralateral muscle activation depending on whether the motor command is excitatory or inhibitory (e.g., Barral et al., 2006, 2010). With training, individuals can often compensate for neural crosstalk that is dispatched to the contralateral limb (Barral et al., 2006, 2010). However, the failure to inhibit, suppress, or otherwise compensate for the neural crosstalk may result in unintended motor actions (Houweling et al., 2010). In addition, it may be more difficult to compensate for this neurophysiological influence in an unfamiliar condition, such as microgravity. As such, it is possible that the integration of contralateral and ipsilateral neural signals during the 1:1 bimanual force task is resulting in increased peak forces in microgravity. However, more research is needed in altered-gravity environments to understand how inherent and incidental constraints influence bimanual coordination dynamics in these environments.

Results for mean force (Figure 6F) also indicated marginally significant (*p* = 0.050) differences during the 1:1 task between the Earth and microgravity conditions, but only for the non-dominant (left) limb. This result is consistent with a number of investigations demonstrating asymmetries between the dominant and non-dominant limb during sensorimotor performance in 1 g (Goble and Brown, 2008). Bimanual asymmetries have been associated with enhanced motor control of the dominant limb/hemisphere to generate motor commands and utilize sensory feedback (de Poel et al., 2007) as well as limb specialization, with the dominant limb being better at controlling dynamic actions and the non-dominant limb being better at stabilizing actions (Sainburg, 2002). Understanding bimanual asymmetries associated with altered-gravity environments may be particularly important considering the divergent role for each limb used for instrument control during spaceflight [e.g., operation of the International Space Station (ISS) robotic arm with multiple degrees of freedom and different type of controllers (rotational vs. translational) for each limb].

Given that 1:1 coordination task is considered to be the central nervous system (CNS) default coordination mode, differences in force production with gravity suggest that the coordination landscape differs between Earth and altered-gravity environments for force-production-related parameters. However, it is important to note that the Lissajous displays provided integrated feedback information regarding the timing of the force pulses. Participants did not receive specific feedback regarding the accuracy of their produced force. It is believed that Lissajous feedback provides the CNS an opportunity to override inherent proprioceptive, vestibular, visual, and/or cognitive constraints acting on the system by providing a “reference of correctness” and information necessary to detect and correct bimanual coordination errors in real-time (Shea et al., 2016). This type of feedback information has proved successful in a number of bimanual coordination experiments, including experiments that required coordination of complex patterns of isometric force pulses (Kennedy et al., 2015, 2016a,b; Wang et al., 2021). In these experiments, participants were able to accurately produce the goal force without additional feedback information. However, in light of the current results in which differences between Earth and microgravity conditions were observed for measures associated with force production, we question whether real-time information regarding force accuracy can be incorporated into the display information, and if this information can be used to counteract force control deficits in altered-gravity environments. Current work in our laboratory is exploring this possibility.

### Retention and Transfer

In the current experiment, participants trained to produce a 1:2 coordination pattern using Lissajous displays to guide performance in an upright position (Earth condition). Note, the 1:1 in-phase task was used as a control condition. To assess whether participants learned to use this feedback to successfully produce the complex goal pattern, a retention test was performed after a short delay period. When we compared performance at the end of training (trial 14, Figure 4) to performance on the retention test for the 1:2 task, the results clearly indicated that participants effectively learned to produce the goal coordination patterns with Lissajous displays. More importantly, to assess whether participants could transfer their training performance during the Earth condition to a new environment (microgravity) with no additional training in the new environment, a transfer test was performed. When we compared performance at the end of training (trial 14, Figure 4) to performance on the transfer test, results clearly indicated that participants could effectively produce the goal coordination pattern in the microgravity condition as well. The transfer of bimanual performance from Earth to microgravity is not trivial, especially given that previous research investigating rhythmic arm movements in microgravity has indicated that the CNS may use different motor control strategies when performing actions in altered-gravity environments compared to when performing the same action under Earth’s gravity (White et al., 2008). In addition, this line of inquiry is particularly relevant given that astronaut training often requires individuals to practice mission relevant tasks on Earth and then apply skills in microgravity.

Previous research has consistently reported that manual control performance is degraded in altered-gravity environments compared to Earth’s gravity, especially during initial exposure (Hermsdörfer et al., 1999; Lackner and DiZio, 2006; Kanas and Manzey, 2008; Clark et al., 2015; Manzey, 2017). In addition, research has reported that dual-tasking of cognitive and motor behavior is significantly impaired during the initial exposure to a microgravity environment (Manzey et al., 1993, 1995, 1998). Note, bimanual coordination is often considered a special case of dual-task performance (Hazeltine et al., 2003). Manual control performance during the first 100–200 s is often critical for many mission critical tasks (Clark et al., 2015). As such, degraded performance during initial exposure to altered-gravity environments is a serious concern. In the current experiment, participants were able to effectively perform the complex bimanual coordination task in microgravity with no training in microgravity. Remarkable, participants only performed two 20 s trials (40 s) and performance was as good as (or better than) that observed during training in the Earth condition. The results of this study suggest that integrated feedback manipulations are a promising countermeasure for sensorimotor decrements observed during microgravity. Similar results were observed in a recent investigation using haptic cues to compensate for sensorimotor impairments in microgravity (Weber et al., 2021). It appears that feedback manipulations may facilitate the successful performance of mission critical tasks, and future work should continue to explore constraints that can facilitate or interfere with bimanual control performance.

### Limitations

To begin to understand the influence of microgravity on bimanual coordination dynamics, we selected a sample population consistent with similar experiments using Lissajous displays to guide bimanual performance in 1 g. The majority of these experiments utilized young (<30 years) male and female participants (e.g., Kovacs et al., 2010a,b; Kovacs and Shea, 2011; Wang et al., 2013, 2021; Kennedy et al., 2016b,^2017^). However, it is important to note that a recent experiment indicated strong effects of age and gender on bimanual force control (Rudisch et al., 2020). While the current experiment was limited to young participants, understanding how the control of bimanual actions in microgravity differs across the lifespan is important, especially given the typical age of astronauts (∼ > 40 years old), and the possibility for individuals of all ages to experience microgravity with the commercialization of spaceflight. Further work is needed to fully understand the impact of age and gender on bimanual coordination dynamics in altered-gravity.

In experiments specifically addressing age-associated changes in the control of bimanual actions, the results generally indicate significantly lower accuracy and higher variability in older adults (> 60 years) compared to young adults (<30 years) (e.g., Vieluf et al., 2015; Rudisch et al., 2020; Roman-Liu and Tokarski, 2021). Note, however, this research did not provide Lissajous displays. A number of mechanisms have been proposed to account for the decline in bimanual performance and learning typically observed in older adults including reduced attentional resources, deficits in cognitive function, and deficits in sensorimotor processing (e.g., Welford et al., 1969; Salthouse et al., 1996; Lorenzo-López et al., 2008). Given that Lissajous displays reduce attentional, cognitive, and/or perceptual demands associated with the task or task environment, it is logical that Lissajous displays may improve bimanual performance in older adults. Indeed, in the limited experiments in which Lissajous displays were provided to older adults to guide bimanual performance, results indicated that they were able to perform the complex bimanual patterns similar to young adults (i.e., interpeak interval ratio, phase angle slope ratio, cycle duration ratio), despite their increased variability (Kennedy and Shea, 2015; Leinen et al., 2016). Consistent with this notion, the results of the current investigation suggest that Lissajous displays may be an effective method to counteract the increased attentional, cognitive, and/or perceptual demands associated with altered-gravity environments. As such, Lissajous displays may prove particularly beneficial for individuals of all ages during spaceflight. In particular, considering that astronauts likely have better coordination than the general population and that they possibly need less training to effectively produce and transfer coordination patterns with Lissajous displays, we expect our results to be strengthen when using this specific population. However, additional research is warranted to confirm this hypothesis.

Finally, while HDT/HUT paradigms are very well established analog to investigate altered-gravity environments (Clement et al., 2015; Diaz Artiles et al., 2016; Diaz-Artiles et al., 2019; Petersen et al., 2021; Whittle et al., 2021), 6° HDT is not a fully accurate representation of microgravity conditions during spaceflight. The presence of a transverse gravitational component (front-to-back, or Gx) and a small longitudinal gravitational component (foot-to-head, or Gz) are limitations inherent to our ground-testing simulation. In addition, our apparatus also does not replicate the potential motion sickness that some astronaut experience when entering in microgravity conditions (Lackner and DiZio, 2006; Diaz-Artiles et al., 2017). Despite these limitations, tilt paradigms reproduce altered-gravity responses reasonably well, and remain part of the most implemented altered-gravity simulations on the ground. However, further efforts should include research in true microgravity conditions, using parabolic flights, the ISS, and/or other commercial opportunities.

## Data Availability Statement

The raw data supporting the conclusions of this article will be made available by the authors, without undue reservation.

## Ethics Statement

The studies involving human participants were reviewed and approved by the Texas A&M University Institutional Review Board (IRB#IRB2020-0068D). The patients/participants provided their written informed consent to participate in this study.

## Author Contributions

AD-A and DK: conceptualization and funding acquisition. YW, MD, and RA: data collection. YW and AD-A: data analysis. AD-A, NK, and DK: manuscript preparation. AD-A, YW, MD, RA, NK, and DK: review, editing, and final approval. All authors contributed to the article and approved the submitted version.

## Conflict of Interest

The authors declare that the research was conducted in the absence of any commercial or financial relationships that could be construed as a potential conflict of interest.

## Publisher’s Note

All claims expressed in this article are solely those of the authors and do not necessarily represent those of their affiliated organizations, or those of the publisher, the editors and the reviewers. Any product that may be evaluated in this article, or claim that may be made by its manufacturer, is not guaranteed or endorsed by the publisher.
